# Enhancement of Plasmonic Performance in Epitaxial Silver at Low Temperature

**DOI:** 10.1038/s41598-017-09402-y

**Published:** 2017-08-21

**Authors:** Liuyang Sun, Chendong Zhang, Chun-Yuan Wang, Ping-Hsiang Su, Matt Zhang, Shangjr Gwo, Chih-Kang Shih, Xiaoqin Li, Yanwen Wu

**Affiliations:** 1 Department of Physics and Center for Complex Quantum Systems, University of Texas at Austin, Austin, TX 78712 USA; 2 Department of Physics, National Tsing-Hua University, Hsinchu, 30013 Taiwan; 3 Department of Physics and Astronomy, University of South Carolina, Columbia, SC 29208 USA

## Abstract

We report longer surface plasmon polariton propagation distance based on crystalline crystal silver at low temperature. Although enhanced plasmonic performance at low temperature has been predicted for a long time, it has not been directly observed on polycrystalline silver films which suffer from significant plasmonic losses due to grain boundaries and rough silver surface. Here we show that longer propagation distance can be achieved with epitaxial silver at low temperature. Importantly, the enhancement at low temperature are consistent across silver films grown with different methods.

## Introduction

For many decades, the measured optical constants for silver has had a wide spread of different values^1–6^. This uncertainty is due to the fact that the common methods for preparing silver films, namely vapor deposition, mostly produce polycrystalline (PC) silver with large variations in quality. In these silver films, grain boundaries, rough surface qualities all contribute to extra scattering and absorption losses which, in turn, can greatly affect the measured values of the optical constants. The inconsistency in the optical constants is a lingering inconvenience for applications utilizing silver structures, such as in the field of plasmonics^7–19^. In order to assess and predict the performance of plasmonic devices, one must then measure the optical constants of the specific PC silver used in each case. This practice is highly unrealistic, especially for nanoparticles where optical constants cannot be readily extracted based on widely used techniques such as spectral ellipsometry. Fortunately, recent advances in silver growth and chemical synthesis have been able to produce high quality single crystalline (SC) silver in both forms of bulk and thin films with relative ease^20–27^. In contrast to PC silver, SC silver possesses atomically smooth surfaces and contains no grain boundaries. In the absence of these external factors, the loss should be dominated by the intrinsic properties of silver, and the associated optical constants should be consistent across different growth and synthesis methods.

In this report, we show that this is indeed the case. We performed temperature dependent surface plasmon polaritons (SPPs) propagation distances (L_SP_) measurements of two SC silver structures grown by two different methods: chemical synthesis^21^ and epitaxial growth^20^. In addition, we compare our results with similar measurements conducted on a SC silver structure produced by a sputtering method^22^. We find that the optical constants of these SC silver are not only consistent across all three silver structures, propagation lengths calculated using these values are also in good agreement with those measured. Furthermore, the propagation distance enhancement ratio shows quantitative agreement between experimental and analytical results. The propagation lengths in all three cases are shown to increase as temperature is decreased, reflecting reduced loss at low temperature as predicted by the electron-phonon scattering model^28, 29^.

## Experiment and Results

**Figure 1:**
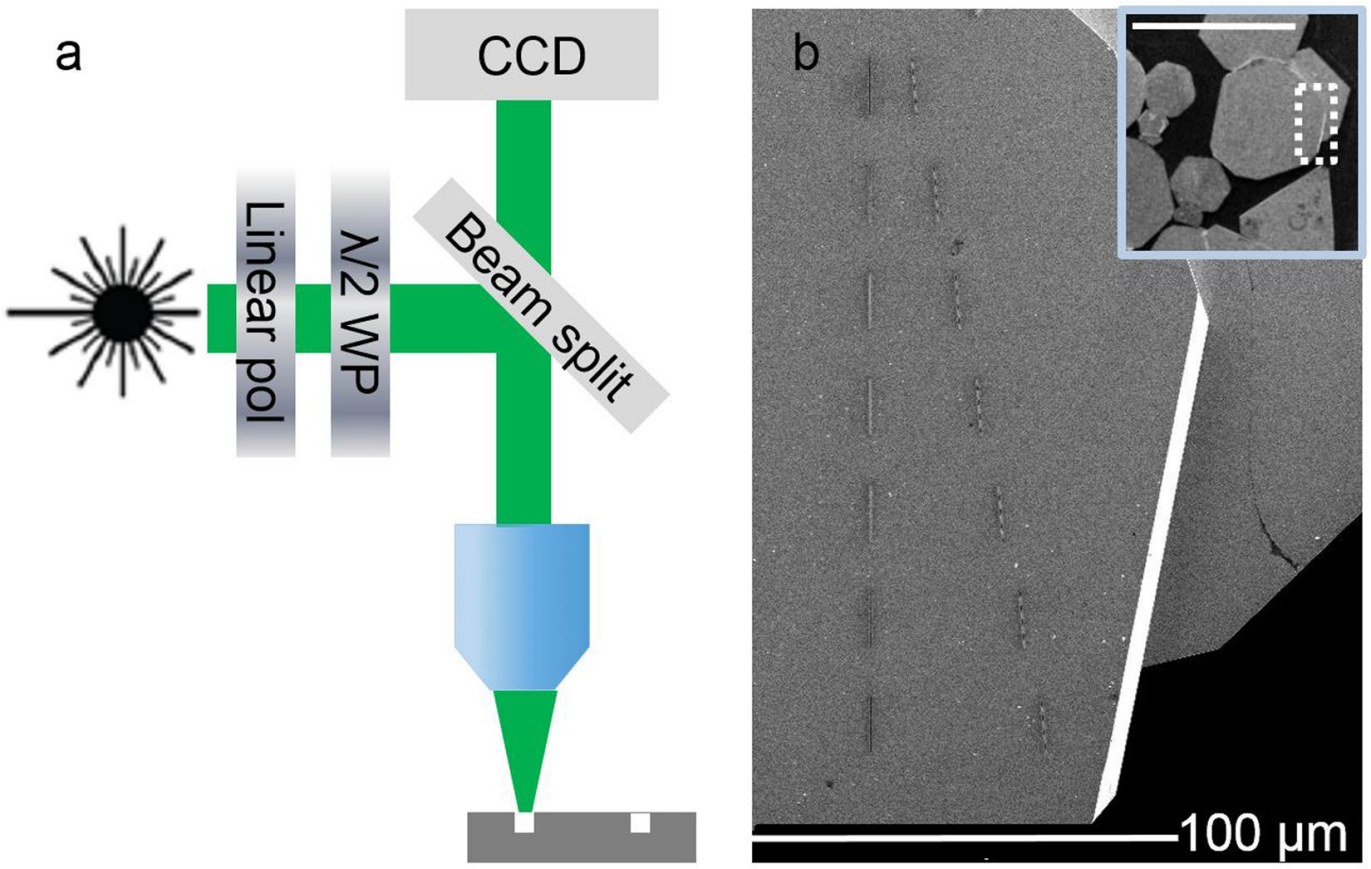
Illustration of experimental setup and SEM image of chemically synthesized silver plates. (**a**) Incident laser at 532 nm is focused on the input groove by an objective lens after a linear polarizer and half waveplate. Scattered SPPs signal from the output groove is collected by the same objective lens and sent to CCD. (**b**) SEM image of the groove pairs. Vertical grooves at left side are input grooves. Output grooves with a 5 degree tilt with r﻿espect to the input grooves are milled at different distances away from the input. Inset is an image of the silver plate at low magnification. Dashed line indicates the area of the main figure. Scale bar in the inset is 500 μ﻿m.

SPPs are electromagnetic waves confined along the interface of metal and dielectric. The propagation distance is related to the total scattering loss in silver and can be calculated directly from the optical constants. SPPs cannot be excited by light in free space on an unstructured metal-dielectric interface due to the momentum mismatch between the light and SPPs^9^. Therefore, we measured the SPPs propagation length experimentally by using a two-groove technique at both room temperature and cryogenic temperature. The setup is illustrated in Fig. 1a. Pairs of grooves (input and output grooves) were milled on the SC silver structures. SPPs were launched by illuminating the input groove with TM-polarized light, where the electric field component of incident light is perpendicular to the groove axis. The SPPs then propagated towards the output groove where they were converted back into light in the free space. The intensity of the out-coupled light was measured by a charge-coupled device (CCD) as a function of the distance between the input and output grooves and fitted with an exponential function. Details of the optical setup can be found in the Method Section.

### Propagation distance on SC colloidal silver

We first measure L_SP_ at room temperature (300 K) with incident light wavelength at 532 nm on a tens of microns thick colloidal SC silver plate grown by chemical synthesis^21, 30^. Synthesis method can be found in the Method Section. As shown in Fig. 1 b, series of groove pairs were milled with increasing distances of 15, 20, 25, 30, 35, 40, and 50 µm (center to center distance between the groove pairs). We note that the input (left side) and output (right side) grooves are not perfectly parallel. An angle of 5 degree is introduced between the groove pair to eliminate SPPs interference from reflections^21^. A 5 nm thick Al_2_O_3_ layer was immediately grown on the silver surface after FIB milling using atomic layer deposition (ALD) to protect the silver structures against oxidation induced degradation.

**Figure 2:**
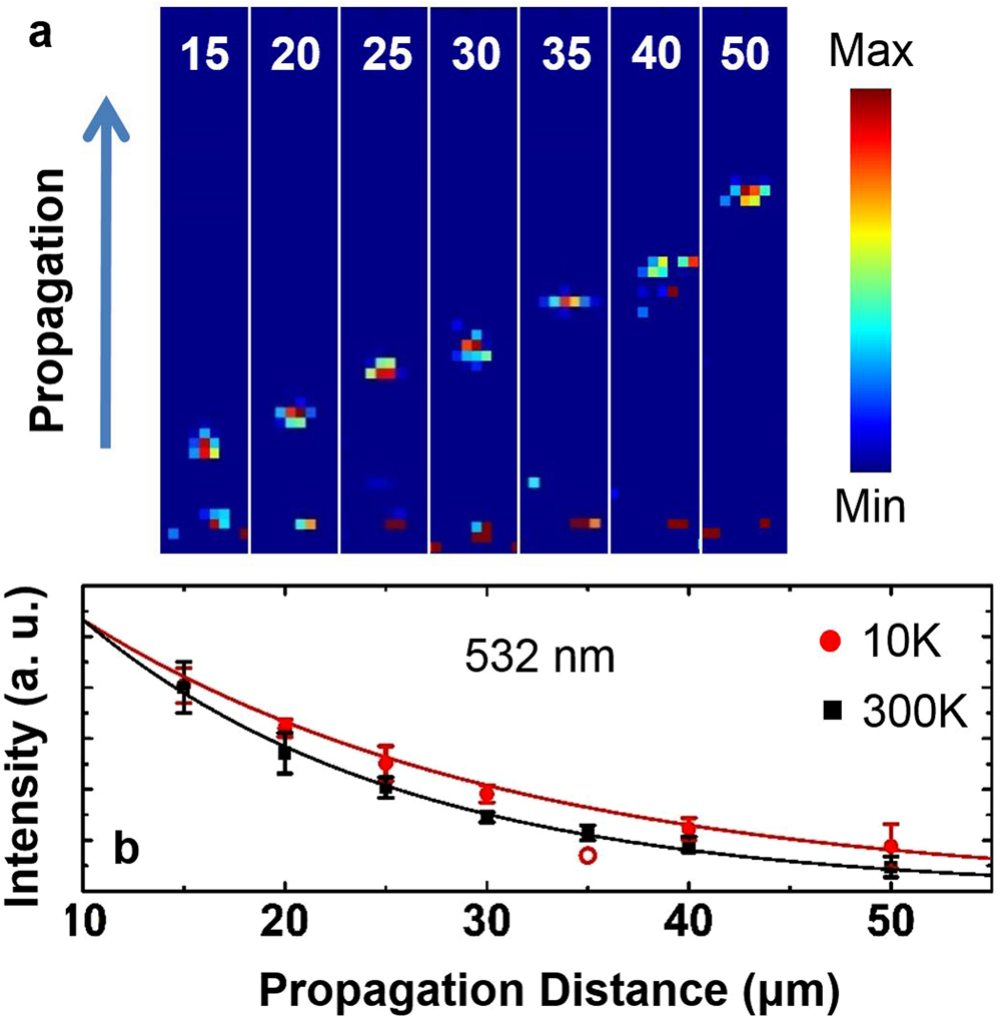
Extraction of propagation distance at 300K with incident light at 532 nm. (**a**) Intensity images captured by CCD. (**b**) Integrated output intensity as a function of distance. Solid black squares and solid red circles are experimental data from 300K and 10 K, respectively. Solid lines are exponential fits. Open red circle is a data point discarded in the fitting due to a small surface crack appeared between that particular groove pair during sample cooling.

Figure 2a shows a set of intensity images from the output grooves used for determining scattered SPPs intensity. All optical measurements are performed with the polarization of the incident electric field oriented perpendicular to the groove axis. Each of the intensity maps was captured using a 0.1 second exposure time with 100 frames average. The integrated output intensities are plotted as square dots in Fig. 2 b. The intensity is fitted with a single exponential decay function and the propagation length is found to be 15.8 ± 0.7 µm. The error bars are extracted from three independent measurements.

In a similar manner, we obtained L_SP_ at a cryogenic temperature of T = 10 K. L_SP_ was found to be 21.3 ± 2.8 µm (Fig. 2 b). The propagation distance of the silver plate exhibits an enhancement of 33% with comparison to that measured at room temperature. During the cooling process, small cracks can develop randomly on the surface of the silver surface. This is due to the mismatch between the thermal expansions of silver and the silicon substrate as well as the internal strain in the silver structure itself. A crack appeared between the groove pair of distance 35 µm. SPPs were scattered by the crack before reaching the output groove. As a result, the intensity obtained from this groove is abnormally low (seen as an empty circle in Fig. 2 b). Therefore, this point was disregarded when extracting propagation distance at low temperature (see supporting material).

### Propagation distance on MBE grown SC silver film

We also examined L_SP_ on a silver film grown by the molecular beam epitaxy (MBE) method. The film we investigated is 45 nm thick grown on a heavily doped Si (111) substrate. The film is capped with 1.5 nm of MgO inside the MBE chamber and 2 nm of Al_2_O_3_ using the ALD immediately after growth. Detail of the MBE growth procedure is shown in the Method Section.

**Figure 3:**
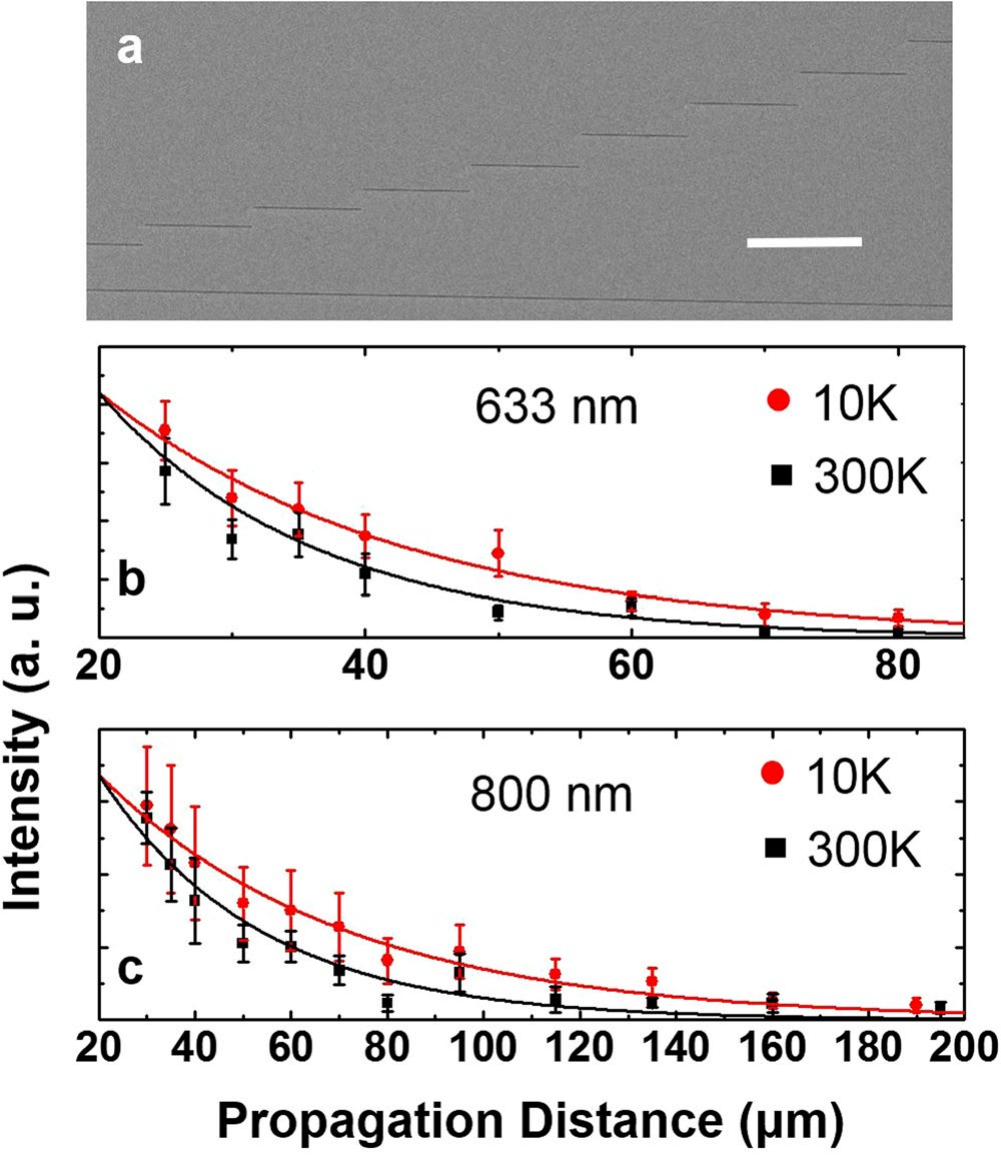
Propagation distance measurements from an MBE grown silver film. (**a**) SEM image of silver film grown with MBE method. Several short input slits are milled with FIB method. A long output slit is milled near the bottom. Scale bar is 20 μm. Integrated output intensity as a function of distance for incident laser of wavelength at (**b**) 633 nm and (**c**) 800 nm. Black squares and red circles are experimental data from 300 K and 10 K, respectively. Solid lines are exponential fittings.

Here we performed the measurement with incident laser wavelength at 633 nm and 800 nm. Because L_SP_ increases at longer wavelength and further enhancement are expected at low temperature, grooves pairs with larger separation were fabricated to ensure our measurement covers the entire range (seen in Fig. 3a). The optical setup is modified slightly because the input and output grooves cannot be captured simultaneously within the field of view of an objective lens (see supporting material). The integrated output intensities are plotted in Fig. 3b and c. At 633 nm illumination, we extracted L_SP_ of 16.1 ± 4.3 µm and 23.1 ± 2.3 µm at 300 K and 10 K, respectively. At 800 nm illumination, we extracted L_SP_ of 33.1 ± 4.9 µm and 50.8 ± 4.0 µm at 300 K and 10 K, respectively. The enhancement is improved by 43% and 51% at 633 nm and 800 nm, respectively.

## Discussion

### Improved propagation distance of SC films over PC films

The mechanisms responsible for SPPs dissipation include several channels: grain boundaries, surface defects, electron-electron scattering, and electron-phonon scattering. Contribution from grain boundary scattering is substantially reduced in the SC silver film^20, 23, 27^. We first calculate the propagation distance based on a two-layer vacuum silver model. In this model, the dispersion relation of SPPs along the vacuum silver interface is given by^9^

1 $${k}_{SPPs}={k}_{0}\sqrt{\frac{{\varepsilon }_{1}{\varepsilon }_{2}}{{\varepsilon }_{1}+{\varepsilon }_{2}}}$$

where ${\varepsilon }_{1}$ and ${\varepsilon }_{2}$ are the permittivity of vacuum and silver, respectively, ${k}_{0}$ the wavevector of incident light in free space. L_SP_ is determined from imaginary part of ${k}_{SPPs}$,

2 $${L}_{SP}={(2Im({k}_{SPPs}))}^{-1}=\frac{1}{2(\omega /c)}{[Im(\sqrt{\frac{{\varepsilon }_{2}}{1+{\varepsilon }_{2}}})]}^{-1}$$

Take propagation distance at a free space wavelength of 532 nm as an example. For our epitaxially grown films, the analytical calculation based on the measured optical constants^20^ predicts a longer propagation distance of 52 µm for a vacuum/metal interface. As comparisons, the calculated propagation distances of template-stripped (TS) and rough PC films are 21.3 µm and 8.7 µm^22^, respectively. This improvement is confirmed in experimentally measured propagation distance as well: the measured propagation distance on SC silver plate is 15.8 µm. Propagation distance on TS and rough PC silver films were reported as about 10 µm and 3 µm, respectively^31, 32^. We note there are discrepancies between experimental and calculated values for all cases. We attribute the discrepancies to two factors. The first reason is the protection layer (MgO and/or Al_2_O_3_) on top of the silver surface. The dielectric layer of higher refractive index than vacuum modifies the SPPs profile at the interface and leads to stronger confinement of SPPs at the interface^33^. Therefore, the propagation distance reduces as a result of the capping layer. We see better agreement between theoretical and experimental results by taking into account the capping layer effect. For example, L_SP_ decreases from 52 µm to 40 µm with a 5 nm Al_2_O_3_ capping layer (see supporting material). However, this effect is negligible for thin capping layers. A second and more dominant reason is the possible degradation and contamination occurred during sample transfer and the FIB milling process. Nevertheless, despite of these imperfection, a value of 15.8 µm at 532 nm for L_SP_ at room temperature still shows an excellent performance. Our results, together with previously reported propagation distance measurement on SC films consistently show improvement over PC films^21, 22, 32^. We also emphasize that although the samples investigated were exposed to ambient environment to varying extents and lengths of time, their performance can still be predicted from the measured optical constants of a separate SC silver film reasonably well^20^.

### Consistent enhancement of propagation at low temperature

The intrinsic L_SP_ depends on the electron mean free path (MFP) and hence the electric conductivity of metals. It is known that at low temperature, noble metals exhibit higher electric conductivity as a result of longer MFP^34–36^. Therefore a smaller imaginary part of dielectric permittivity of metals would be expected at lower temperature according to the Drude model^37, 38^. However, only very limited measurements have been carried out with emphasis on the temperature dependence of the optical constant of plasmonic metals^39–41^. Even less efforts were made to explore SPPs propagation distance at low temperature^42^. This is mostly due to the fact that the accuracy of the measurement depends highly on the quality of silver films. In other words, if the effect from the electron-phonon scattering is overwhelmed by other extrinsic decay channels, such as electron-grain boundary and electron-defects scatterings, the temperature dependent effect will not be measurable^42^.

SC silver film, which has less electron- grain boundary scattering, provides the possibility to explore the enhancement of propagation distance at low temperature. In our experimental results on SC silver structure, we obtain L_SP_ enhancement of 33%, 43% and 51% at 532 nm, 633 nm and 800 nm, respectively, from 300 K to 10 K. We compare our results with that from another SC silver film grown on mica substrates by dc magnetron sputtering at maximum deposition rate and high temperature^23^ and studied by Jayanti and his colleagues^22^. The comparison results are summarized in Table 1. Remarkably, a quantitative agreement is achieved between the enhancements observed in our experimental results and those calculated from the temperature dependent optical constants (see Table 1) reported in ref. 22. In their work, enhancement of 38% and 45% were expected for 500 nm to 633 nm, respectively, from room temperature to 25 K. The propagation distance enhancement at low temperature is a convincing evidence that SC films provide better platforms for SPPs propagation and subsequently for plasmonic applications.

**Table 1:** Summary of the *L*_SP_ (unit in µm) and the enhancement at low temperature.

532 nm			633 nm			800 nm		
RT	Cryo	Enhance	RT	Cryo	Enhance	RT	Cryo	Enhance
15.8*	21.3*	34%*	16.1^†^	23.1^†^	43%^†^	33.1^†^	50.8^†^	53%^†^
9.4^‡^	10.3^‡^	12%^‡^	12.7^‡^	19.3^‡^	52%^‡^			
25.7^§^	35.5^§^	38%^§^	61.4^§^	89.6^§^	45%^§^	142^§^	218^§^	55%^§^

*Chemical Expt. ^†^MBE Expt ^‡^Sputtering Expt ^§^Sputtering Theory.

## Conclusion

In conclusion, we measured SPPs propagation distance on SC silver films grown with MBE and chemical synthesis methods. The propagation distances measured reflect consistent improvement when compared to that of PC silver films. In addition, we experimentally observed 30% to 50% enhancement of SPPs propagation distances at low temperature on SC silver films. These enhancements show consistency from SC silver films grown and measured by different groups, which indicates that we are observing nearly intrinsic optical properties of silver. These results are useful for future design of plasmonic devices and should alleviate the uncertainty regarding the predictability of device performance involving silver structures.

## Method

### Sample 1 Colloidal silver plate preparation

The method of synthesizing giant single crystalline silver plates is based on a modified platinum-catalyzed, ammonium hydroxide (NH_4_OH)-controlled polyol reduction method^30^. To obtain silver crystal of larger size, the concentration of NH_4_OH is increased for a much slower reaction rate. Here, NH_4_OH plays the role of a stabilizer in the reduction process. In the polyol process, controlling the nucleation and growth steps in the reaction media is important for the silver crystal size and shape. The reaction takes 5 days. After that, silver plates with millimeter-scale lateral sizes appear on the bottom or sidewall of the glass container while the reaction media remain clear

### Sample 2 MBE silver film preparation

The films are grown on heavily doped Si(111)-7 × 7 substrates using the established “two-step” method. First, 20 monolayer silver is evaporated onto a Si(111) substrate held at low temperature (~ 90 K). The deposition rate is pre-calibrated by a quartz crystal monitor as ~1 Å/s, and a commercial Knudsen cell is used to maintain the stable deposition during the growth. Secondly, the sample is naturally annealed to room temperature over a time period of no less than five hours. This two-step process is repeated until a desired thickness is obtained. An amorphous MgO capping layer is deposited *in-situ* after the silver growth to prevent oxidation and de-wetting of silver films. The sample is further coated with a 2 nm Al_2_O_3_ capping layer using an atomic layer deposition (ALD) method immediately upon removal from the MBE chamber^20^.

### Optical measurement

A single mode fiber coupled, continuous-wave laser is used to excite SPPs. The polarization of the laser is controlled by a fixed linear polarizer (CVI CLPA-8.0) combined with a half wave plate (WPH10M-532). The incident laser is focused onto the input grooves by an 20× objective lens (50× objective lens for measurements on MBE grown film) after reflected by a beam splitter (Thorlabs BS025, T: R = 90: 10). The decoupled SPPs signal is collected by the same objective and transmitted through the beam splitter and focused on a CCD (Andor Newton920, pixel size 26 by 26 μm^2^). The focused laser beam has a spot size of about 1 µm, determined by an image of the reflected light from flat silver film surface. To study temperature dependence of the propagation distance, our sample is loaded in a liquid Helium cooled cryostat system regulated by temperature controller (Cryo-con Model 32B). The out coupled light is quantified by integrating the intensity from the pixels covering the output groove. In order to maximize coupling efficiency, after coarsely tuning the sample translation stage to overlap the input groove and incident beam spot, we finely tune the incident beam direction to optimize the output signal. To eliminate any possible direct scattering from the output groove as well as background signal, we subtracte light intensity collected with incident light polarized parallel to the groove axis within the same integration window.

### Data availability

The datasets generated during and/or analyzed during the current study are available from the corresponding author.

## Electronic supplementary material

Supplementary information
