# Supplementary material for: Enhancement of Plasmonic Performance in Epitaxial Silver at Low Temperature
Source: Sci Rep. 2017 Aug 21;7:8917. doi: 10.1038/s41598-017-09402-y (PMC5567193; doi:10.1038/s41598-017-09402-y)
Supplement: Supplementary file 1 — Supplementary information [file 41598_2017_9402_MOESM1_ESM.pdf]

Supporting Information: Enhancement of Plasmonic Performance in Epitaxial Silver at Low Temperature

Liuyang Sun[1], Chendong Zhang[1], Chun-Yuan Wang[2], Ping-Hsiang Su[1], Matt Zhang[1], Shangjr Gwo[2], Chih-Kang Shih[1], Xiaoqin Li[1], Yanwen Wu[1,3]*

1. Department of Physics and Center for Complex Quantum Systems, University of Texas at Austin, Austin, TX 78712
2. Department of Physics, National Tsing-Hua University, Hsinchu, Taiwan 30013
3. Department of Physics, University of South Carolina, Columbia, SC 29208

E-mail: wu223@mailbox.sc.edu

S1. Intensity Images at 10 K

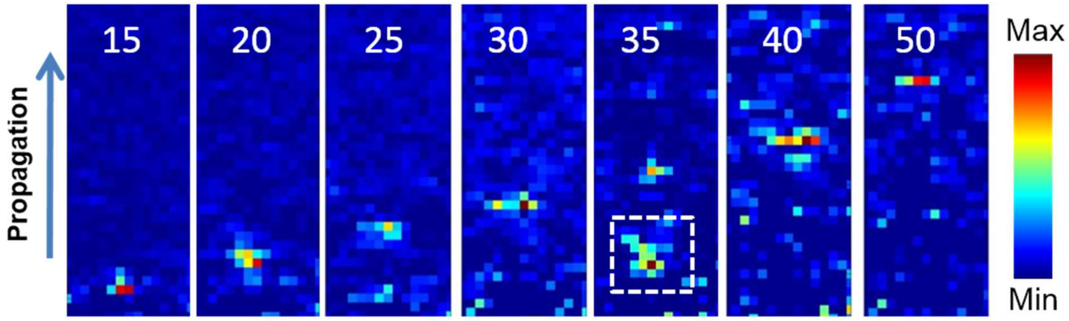

Figure S1. The scattered intensity distribution under excitation at 532 nm. The numbers in the figure indicate distance of the groove pairs in each panels with unit of µm. For the 35 µm groove pair, white box indicates the location of the crack on the silver surface.

During the process of cooling down, cracks can develop randomly on the silver surface. This is due to the thermal expansion mismatch between the silver, silicon substrate, and the capping layer. The internal strain in the silver structure itself also contributes to the formation of cracks. These cracks only affect our measurement when they are between the input and output grooves. The cracks can prematurely scatter the SPPs into free space before they reach the output groove. This is the case for the 35 µm groove pair. In the 35 µm panel illustrated in Fig. S1, we see a bright spot indicated in the white dashed line box

due to a crack. As a result, the intensity count from the output groove is significant lower than expected without the influence of this crack. Therefore, we have removed this data point when we perform the exponential decay fitting of the propagation length on this particular case..

S2. Optical Setup for measuring SPPs propagation distance on molecular beam epitaxy grown silver film

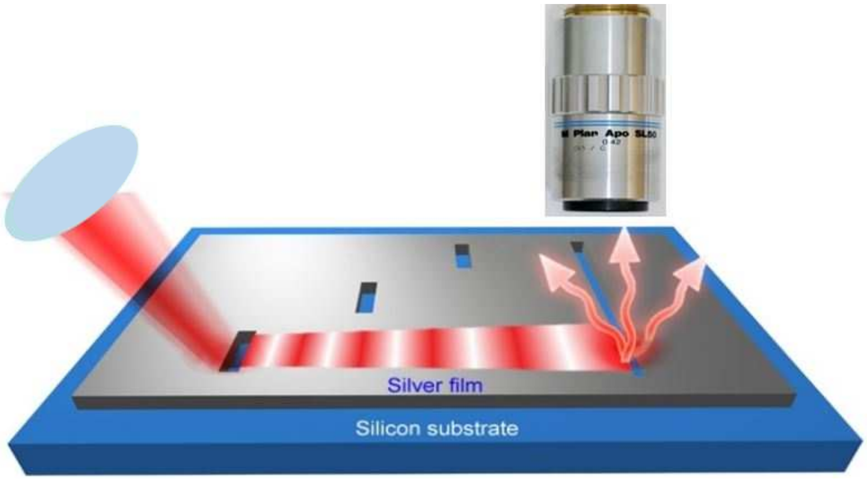

Figure S2. Illustration of experimental setup.

For propagation distance measurement at wavelength 633nm and 800nm, grooves pairs with larger separation were fabricated to ensure our measurement covers longer propagation distance. As a result, the input and output grooves cannot be captured simultaneously within the field of view of one objective lens. Therefore, the optical setup is modified slightly comparing to the setup shown in main text Figure 1a. As illustrated in Figure S2, we focused incident laser through an aspherical lens onto input grooves, and collect the scattered SPPs signal with a 50X objective.

S3. Intensity maps acquired at 633 nm and 800nm

Using the modified setup, we measured the intensity distribution at output groove. Figure S3 and S4 shows the intensity acquired with incident light at wavelength 633 nm and 800 nm, respectively. The top and bottom panels of each figure is the intensity distribution at room temperature and 10K, respectively.

In the modified setup as mentioned in supporting section S2, the output grooves is centered at the field of view of the collecting objective lens.

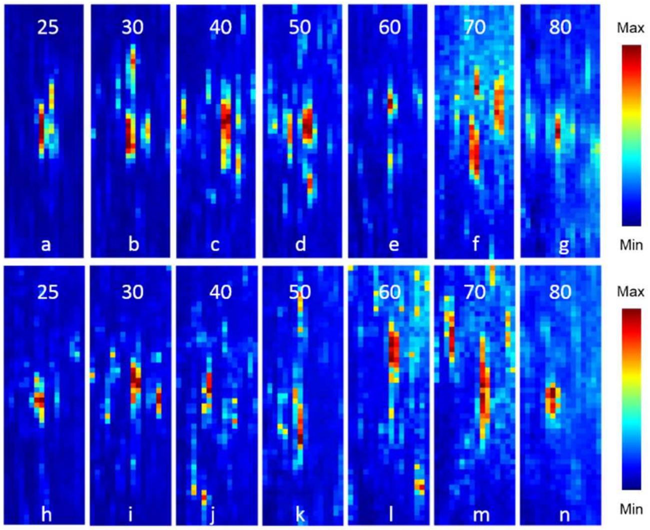

Figure S3. The scattered intensity distribution under excitation at 633 nm. Top and bottom panel are acquired at room temperature and 10 K, respectively.

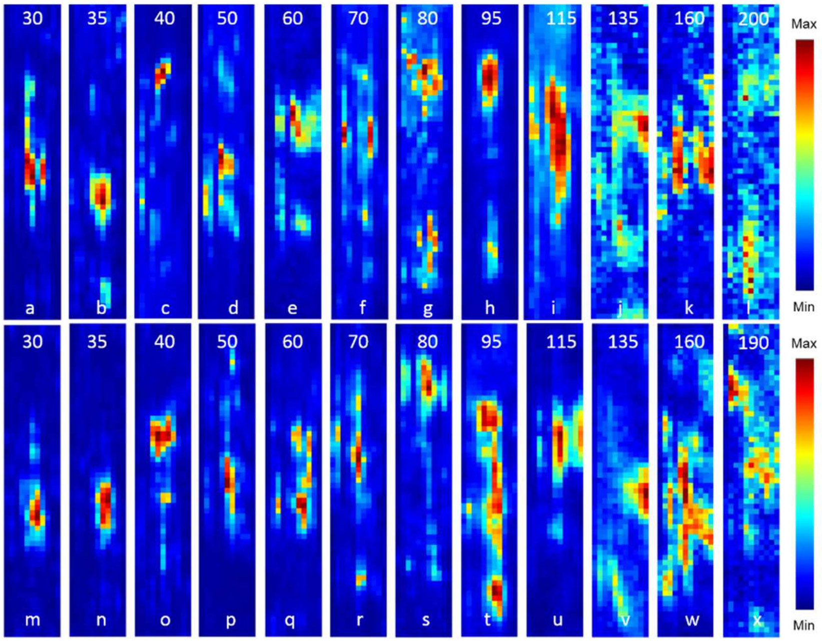

Figure S4. The scattered intensity distribution under excitation at 800 nm. Top and bottom panel are acquired at room temperature and 10 K, respectively.

S4. Effect of capping layer

Experimentally a dielectric layer was grown on top of silver film after FIB milling to protect the silver from oxidation. In main text we didn't include the capping layer in the two-layer model. Here we examine the effect from capping layer by performing numerical calculation based on finite-difference time-domain method. Figure S5 shows numerical calculation taking account the Al_2O_3 capping layer. Over the wavelength range we calculated, L_{SP} reduces as thickness of the Al_2O_3 increases.

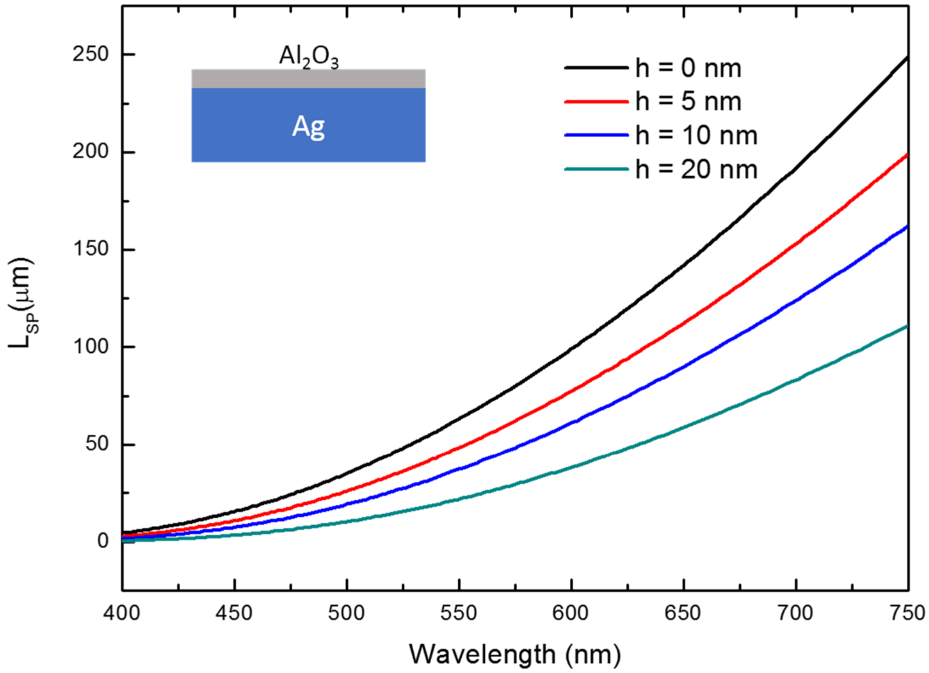

Figure S5. Numerical calculated propagation distance. The inset shows the model we consider. Propagation distance with Al_2O_3 thickness (h) of 0, 5, 10, and 20 nm are shown as black, red, blue and green curve, respectively.
